# Distinct Oncogenic Transcriptomes in Human Mammary Epithelial Cells Infected With Cytomegalovirus

**DOI:** 10.3389/fimmu.2021.772160

**Published:** 2021-12-22

**Authors:** Sandy Haidar Ahmad, Sébastien Pasquereau, Ranim El Baba, Zeina Nehme, Clara Lewandowski, Georges Herbein

**Affiliations:** ^1^ Pathogens & Inflammation/EPILAB Laboratory, EA4266, Université de Franche-Comté, Université Bourgogne Franche-Comté (UBFC), Besançon, France; ^2^ Department of Virology, Centre Hospitalier Universitaire (CHU) Besançon, Besançon, France

**Keywords:** HCMV, cytomegalovirus, oncogenesis, breast cancer, high-risk, low-risk

## Abstract

Human cytomegalovirus is being recognized as a potential oncovirus beside its oncomodulation role. We previously isolated two clinical isolates, HCMV-DB (KT959235) and HCMV-BL (MW980585), which in primary human mammary epithelial cells promoted oncogenic molecular pathways, established anchorage-independent growth *in vitro*, and produced tumorigenicity in mice models, therefore named high-risk oncogenic strains. In contrast, other clinical HCMV strains such as HCMV-FS, KM, and SC did not trigger such traits, therefore named low-risk oncogenic strains. In this study, we compared high-risk oncogenic HCMV-DB and BL strains (high-risk) with low-risk oncogenic strains HCMV-FS, KM, and SC (low-risk) additionally to the prototypic HCMV-TB40/E, knowing that all strains infect HMECs *in vitro*. Numerous pro-oncogenic features including enhanced expression of oncogenes, cell survival, proliferation, and epithelial-mesenchymal transition genes were observed with HCMV-BL. *In vitro*, mammosphere formation was observed only in high-risk strains. HCMV-TB40/E showed an intermediate transcriptome landscape with limited mammosphere formation. Since we observed that Ki67 gene expression allows us to discriminate between high and low-risk HCMV strains *in vitro*, we further tested its expression *in vivo*. Among HCMV-positive breast cancer biopsies, we only detected high expression of the Ki67 gene in basal tumors which may correspond to the presence of high-risk HCMV strains within tumors. Altogether, the transcriptome of HMECs infected with HCMV clinical isolates displays an “oncogenic gradient” where high-risk strains specifically induce a prooncogenic environment which might participate in breast cancer development.

## Introduction

Breast cancer is the most common cancer diagnosed among women and recognized as one of the main causes of death in women (1). Etiologic factors of breast cancer are classified into genetic and environmental risk factors (2), and among these latter viruses could be involved (3, 4). Furthermore, the DNA and gene products of several viruses have been identified in breast cancer biopsies (5). Human cytomegalovirus (HCMV), a ubiquitous pathogen belonging to the *Herpesviridae* family, has been detected in 90% of early and metastatic breast cancers (6–8). Likewise, HCMV DNA or antigens have been found in other malignancies including brain (glioblastoma, medulloblastoma), colon, prostate, and liver (7, 9–13). HCMV causes asymptomatic to mild infection in immunocompetent host, but it can lead to serious complications in the immunocompromised host and cancer patients (14, 15).

Although the growth of laboratory HCMV strains is restricted to fibroblasts, the clinical HCMV isolates infect a broad range of cells including epithelial cells, endothelial cells, monocytes, macrophages, fibroblasts, stromal cells, hepatocytes, smooth muscle cells, and neural stem/progenitor cells (16–19). Despite its known onco- and immunomodulatory effects, HCMV can transform primary human mammary epithelial cells (HMECs) *in vitro* as previously reported for the HCMV-DB and HCMV-BL strains (20, 21) in addition to the human embryonal lung fibroblasts (22). Moreover, blood monocyte, tissue macrophages, CD34+, and neural stem/progenitor are identified as HCMV reservoirs harboring latent virus (17, 23–28).

The different oncogenic abilities, cellular and molecular characteristics allowed us to classify HCMV strains into high or low-risk oncogenic strains. The high-risk strains promoted oncogenic molecular pathways, induced the expression of stemness markers, transformed epithelial cells, induced the appearance of polyploid giant cancer cells (PGCCs) in culture, established anchorage-independent growth *in vitro*, and produced tumorigenicity in mice models (20, 21). In contrast, the low-risk strains did not display such oncogenic features (21).

Herein, we compared six clinical HCMV strains for their oncogenic potential in HMECs using soft agar assay and transcriptomic analysis. Therefore, we compared the transcriptome profile of HMECs infected with the high-risk HCMV-BL strain versus the low-risk HCMV-FS, KM and SC strains. The transcriptome of HMECs infected with HCMV-BL presents oncogenic traits, favors cell cycling, cell proliferation, and epithelial-to-mesenchymal transition (EMT). However, the transcriptome of the HCMV-FS, KM and SC low-risk strains did not show any of the previously mentioned characteristics. HMECs infected with HCMV-TB40/E displayed a transcriptome phenotype similar to that of the HCMV-FS low-risk strain. In agreement with these distinct transcriptome profiles observed, we noticed the highest transformation and mammosphere formation in HMECs infected with the HCMV-BL high-risk strain, but not with the HCMV-FS, HCMV-KM and HCMV-SC low-risk ones. In line with the transcriptome phenotype observed in acutely infected HMECs, we perceived the preferential detection of high-risk HCMV strains along with the high expression of Ki67 in breast cancer biopsies, especially in the basal-like ones.

## Materials and Methods

### Cell Cultures

HMECs were purchased from Life Technologies (Carlsbad, CA, USA). MDA-MB231 and MCF7 cells were provided by Institut Hiscia (Arlesheim, Switzerland). Human embryonic lung fibroblasts (MRC5) were purchased from RD-Biotech (Besançon, France). HMECs were cultivated in HMEC medium (Life Technologies) supplemented with HMEC supplement and bovine pituitary extract (Life Technologies). MDA-MB231, MCF7, and MRC5 were maintained in Dulbecco’s modified Eagle medium (PAN- Biotech) supplemented with fetal bovine serum (Dutscher) and penicillin- streptomycin (Life Technologies). Cultures were free of mycoplasma (VenorGem classic mycoplasma detection, Minerva biolabs).

### HMECs Infection With HCMV

HMECs were infected with HCMV-DB (KT959235), HCMV-BL (MW980585), HCMV-TB40/E (KF297339), HCMV-FS, HCMV-KM, and HCMV-SC. The HCMV clinical isolates were previously isolated in our laboratory (20, 21), except for HCMV-TB40/E (29). Cell-free virus stocks of HCMV were grown in HMECs as described previously (21). Virus titers were determined through the quantification of HCMV load by real-time qPCR detection using KAPA SYBR FAST Master Mix, Omega Bio-TEK) and *IE1* gene primers (sense, 5’-CGACGTTCCTGCAGACTATG-3’; anti-sense, 5’-TCCTCGGTCACTTGTTCAAA-3’) according to the manufacturer’s protocol. Results were collected and analyzed using MxPro qPCR software.

### Soft Agar Colony Formation Assay

Colony formation in soft agar seeded with HMECs uninfected, infected with the six strains listed above, MCF7 cells and MDA-MB231 cells were assayed using Cell Biolabs Cytosolic Cell Transformation Assay kit (Colorimetric assay, CB135; Cell Biolabs Inc., San Diego, CA) as per the manufacturer’s protocol. The detection and quantification of colony formation was assessed by MTT assay (Cayman Chemical, Ann Arbor, MI). Colonies were observed under an Olympus microscope (Olympus Corporation, Tokyo, Japan)

### Confocal Microscopy and Immunohistochemistry

Confocal microscopy was performed as described previously (21). Cells were washed using 1X PBS then fixed and permeabilized using BD Cytofix/Cytoperm, and finally stained with anti-IE1 (pp72) antibody (ab53495, Abcam). To visualize the nucleus, DAPI (4′, 6′-diamidino-2- phenylindole) staining was performed according to the manufacturer’s protocol. Post-staining, confocal images were taken using 63X oil immersion objective lens with a LSM800 Carl-Zeiss confocal microscope 95 (Germany). The Ki67 immunohistochemistry (IHC) evaluation on breast biopsies was assessed using the monoclonal antibody MIB1 (Invitrogen).

### Flow Cytometry Analysis

Uninfected HMECs and HCMV-infected HMECs were fixed and permeabilized for 20 minutes at 4°C using 100 μl Cytofix/Cytoperm solution (BD Biosciences), washed twice with 1X PBS supplemented with 3% FBS and 0.1% Triton-X, and then re-suspended in 100ul of staining buffer (1X PBS, 3% FBS, and 0.1% Triton-X). Cell staining was done using the anti-IE1 (CMV-pp72) antibody conjugated to PE (sc-69834, Santa Cruz Biotechnology). HMECs were incubated in the dark for 60 minutes at 4°C. Cells were washed twice with 1X PBS supplemented with 3% FBS and 0.1% Triton-X and subjected to cytofluorometric analysis on a BD LSRFortessa X-20 (BD Biosciences) flow cytometer. Data obtained was analyzed and processed using FACSDiva software (BD Biosciences).

### Phylogenetic Analysis

Phylogenetic analysis was performed on several HCMV strains [BL (accession number: MW980585); DB (accession number: KT959235); TB40/E (accession number: KF297339); and Davis (accession number: JX512198.1)]. Multi-sequence alignments (MSA) were implemented using CLUSTAL W. Phylogenetic tree was constructed by means of the neighbor-joining method. The conducted analysis was done using MEGA7 software (http://www.megasoftware.net/).

### RT^2^ Profiler™ PCR Arrays Experiment

Total RNA was extracted from uninfected HMECs and HMECs infected with HCMV-BL, HCMV-TB40/E, HCMV-FS, HCMV-KM and HCMV-SC at MOI of 1 at day 1 post-infection using an RNA extraction kit (EZNA Total kit I, Omega BIO-TEK). Then, the cDNA was synthesized using the RT^2^ First Strand Kit (Qiagen, Germantown, MD, USA) following the manufacturer’s instructions. Two RT^2^ Profiler™ PCR Arrays for human breast cancer (PAHS-131ZA) and human oncogenes & tumor suppressor genes (PAHS-502ZR) (both from Qiagen, Germantown, MD, USA) were performed using Mx3005P real-time PCR system as per manufacturer’s instructions. These microarrays allow the quantification of mRNA from 84 cellular genes, using qPCR with premixed primer sets and SybrGreen qPCR MasterMix. DNA contamination control, housekeeping genes, reverse transcription control, and positive control were included in each PCR array. Data were analyzed using the manufacturer web-based analysis software (http://pcrdataanalysis.sabiosciencescom/pcr/arrayanalysis.php). The analysis was done based on three independent biological experiments for each of the HCMV strains.

### Mammosphere Assay

Mammosphere formation by uninfected HMECs and HMECs infected with HCMV-BL, HCMV-TB40/E, HCMV-FS, and HCMV-KM at MOI of 1 was assayed as described previously (19). MCF7 and MDA-MB231 were used as positive controls. Mammospheres larger than 60 microns were counted.

### Western Blotting

Cellular extracts of uninfected and infected HMECs with HCMV-DB and HCMV-TB40/E were prepared at 1 hour, 5 hours, days 1 and 3 post-infection and used to assess the expression of Rb, pRb, Akt, pAkt-Th308, pAkt-Ser473, STAT3, pSTAT3, CyclinD1, and ATM as described previously (20). Protein levels were quantified using ImageJ 1.40 software (National Institutes of Health, Bethesda, MA, USA). β-actin was used as loading control to normalize sample loading. Anti-Rb, anti-pRb, anti-Akt, anti-pAktThr308, anti-pAktSer473, anti-cyclin D1, and anti-ATM antibodies were purchased from Cell signaling (Danvers, MA, USA). Anti-pSTAT3 and anti-STAT3 antibodies were purchased from Santa Cruz Biotechnology (Santa Cruz, CA). Anti-β-actin antibody was purchased from Sigma-Aldrich (St. Louis, MO, USA).

### Breast Biopsies

Nineteen frozen tumor breast biopsies (ten luminal tumor biopsies and nine basal tumor biopsies) and eight adjacent healthy breast biopsies were provided by the regional tumor bank (BB0033-00024 Tumorothèque Régionale de Franche-Comté). Healthy breast biopsies were numbered from one to eight, luminal tumor biopsies from nine to eighteen, and basal tumor biopsies from nineteen to twenty-seven. A detailed histopathological biopsy data including the histologic biopsy type, Elston-Ellis grading system, hormone percentages (estrogen and progesterone), presence or absence of the human epidermal growth factor receptor 2 (HER2) protein, vascular emboli, and TNM staging was provided as a supplementary data (**Supplementary Table 1**). Additionally, using twenty different breast tumor biopsies, we assessed the Ki67 mRNA and Ki67 protein levels measured by RT-qPCR and IHC, respectively, on the same biopsy sample. A written informed consent for participation was obtained from all patients. The study was authorized by the local ethics committees of Besançon University Hospital (Besançon, France) and the French Research Ministry (AC-2015-2496, CNIL n°1173545, NF-S-138 96900 n°F2015). The presence of HCMV, within all the biopsies, was assessed by qPCR using IE1 and UL69 primers as mentioned above and described previously (30). For Ki67 and GAPDH mRNA quantification, RNA was extracted from the biopsies using E.Z.N.A.^®^ Total RNA Kit I (Omega BIO-TEK, GA, USA). Reverse transcription was performed using the SuperScript IV First-Strand Synthesis kit (Invitrogen, Carlsbad, CA, USA). The expression of Ki67 and GAPDH was assessed by real-time qPCR using KAPA SYBR FAST Master mix (KAPA BIOSYSTEMS, KK4601) and specific primer for Ki67 (sense, 5’TCCTTTGGTGGGCACCTAA GACCTG3’; anti-sense, 5’TGATGGTTGAGGTCGTTCCTTG ATG3’) and GAPDH (sense, 5’CCCCTCTTCAAGGCCTC TAC3’; anti-sense, 5’CGACCACTTTGTCAAGCTCA3’) according to the manufacturer’s protocol. The fold change expression was calculated using the delta-delta Ct method (31). Biopsies having a fold change for Ki67 gene expression <30, from 30 to 100, and >100 were considered to have a low, intermediate, and high Ki67 mRNA absolute level respectively. The Ki67 mRNA cut-off was set based on the state of healthy biopsies (32).

### Statistical Analysis

Statistical analysis was performed using the Wilcoxon test with the help of the Mathematics Department of the University of Franche-Comté. *p-value* ≤ *0.05* were considered statistically significant. In data analysis, Microsoft Excel was used to prepare the plots with standard deviation and p-values. Statistical significance was represented on figures by stars, with the following code *p-value<0.05; **p-value<0.01; ***p-value<0.001.

## Results

### Growth and Transforming Capacity of Six HCMV Clinical Strains in HMECs

A phylogenetic analysis was performed to analyse the genomic sequences of four HCMV strains, namely HCMV-DB, HCMV-BL, HCMV-TB40/E, and HCMV-Davis. Since the clinical isolates FS, KM and SC genomes were not sequenced, we selected the strain Davis for its similar clinical characteristics (33). The phylogenetic analysis showed that HCMV-BL is close to HCMV-DB, and HCMV-TB40/E is close to the genomes of HCMV-Davis (**Supplementary Figure 1**). The two observable brackets illustrate a high-risk group including DB and BL strains, and a low-risk group including TB40/E and Davis strains (**Supplementary Figure 1**).

**Figure 1:**
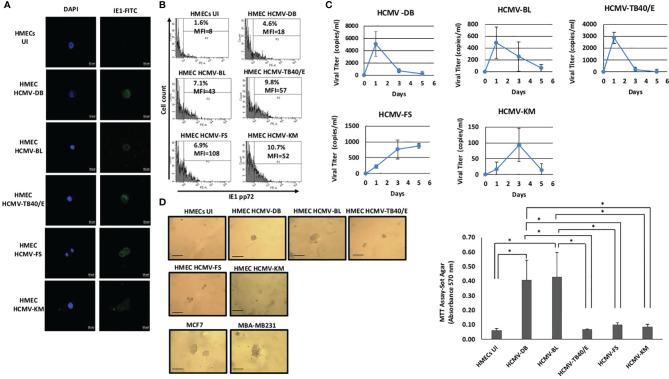
Replication and transforming capacities of five HCMV strains in HMEC cultures. **(A)** Confocal microscopic images of DAPI and IE1 staining in HMECs infected with HCMV-DB, HCMV-BL, HCMV-TB40/E, HCMV-FS, and HCMV-KM. Uninfected HMECs (UI) was used as negative control. Magnification X63, scale bar 10 μm. Data shown are representative of three independent experiments. **(B)** Detection of IE1 (pp72) protein in HMECs infected with HCMV-DB, HCMV-BL, HCMV-TB40/E, HCMV-FS, and HCMV-KM. Uninfected HMECs (UI) was used as negative control. Data are represented as mean ± SD of two independent experiments. **(C)** Growth kinetics of the five HCMV strains in HMECs up to day 5 post-infection. Measurement of IE1 by qPCR in order to assess the viral growth. Data are represented as mean ± SD of three independent experiments. **(D)** Colony formation in soft agar seeded with HMECs infected with HCMV-DB, HCMV-BL, HCMV-TB40/E, HCMV-FS, and HCMV-KM at MOI of 1. MDA-MB231 and MCF7 were used as positive controls. Uninfected HMECs were used as negative control. Colonies were observed under an inverted light microscope (magnification ×100, scale bar 100µm). *Right panel.* Quantification was performed at day 15 post-seeding. Histogram represents mean ± SD of three independent experiments. *p-values* were determined by Wilcoxon test. *p-value < 0.05.

We assessed the replication of six HCMV clinical isolates in HMECs, namely the HCMV-BL and HCMV-DB high-risk strains, the HCMV-FS, HCMV-KM and HCMV-SC low-risk strains, and the prototypic TB40/E strain (20, 21, 29). We evaluated the expression of the immediate early protein IE1 by immunofluorescence and flow cytometry. We observed strong IE1 expression in HMECs infected with the five strains (HCMV-DB, BL, TB40/E, FS and KM) at day 1 post-infection compared to the uninfected HMECs by confocal microscopy (**Figure 1A**). Moreover, the expression of IE1 protein in HMECs infected with the different HCMV strains was confirmed by flow cytometric analysis (**Figure 1B**). Further, we noticed productive replication of all six HCMV strains in infected HMECs as assayed by *IE1* detection in culture supernatants using qPCR (**Figure 1C** and **Supplementary Figure 2A**). An early and transient peak of viral replication was observed at day 1 post-infection for HMECs infected with HCMV-DB, HCMV-BL, and HCMV-TB40/E unlike the delayed peaks at day 3 and 5 post-infection for HMECs infected with HCMV-KM and HCMV-FS respectively. To further assess the oncogenic status of all six HCMV strains, we performed a soft agar assay. HMECs infected with the six HCMV strains were seeded in soft agar at day 1 post-infection. Cancer cell lines, MCF7 and MDA-MB231, were used as positive controls. Uninfected HMECs were used as negative control. Colony formation was detected at day 15 post-infection only with HCMV-DB and HCMV-BL compared to the uninfected HMECs (*p-value*_(HCMV-DB : UI HMEC)_ =0.03, *p-value*_(HCMV-BL : UI HMEC)_ =0.03, *p-value*_(HCMV-DB : HCMV-TB40/E)_ =0.03; *p-value*_(HCMV-DB : HCMV-FS)_ =0.03; *p-value*_(HCMV-DB : HCMV-KM)_ =0.03; *p-value*_(HCMV-BL : HCMV-TB40/E)_ =0.03; *p-value*_(HCMV-BL : HCMV-FS)_ =0.03; *p-value*_(HCMV-BL : HCMV-KM)_ =0.03). In contrast, no significant colony formation was observed with HCMV-FS, HCMV-KM, HCMV-SC, and HCMV-TB40/E infected HMECs (**Figure 1D** and **Supplementary Figure 2B**). Our results indicate that HMECs infected with the high-risk strains BL and DB have an anchorage-independent growth ability.

### Distinct Transcriptome Profiles in HMECs Infected With Low-Risk and High-Risk HCMV Strains

We decided to compare the transcriptome profile of the high-risk BL strain, the low-risk FS, KM and SC strains, and the prototypic TB40/E strain. Human breast cancer along with human oncogenes and tumor suppressor genes RT^2^ profiler PCR arrays were used to screen the infected HMECs with the four strains at MOI of 1 at day 1 post-infection and the uninfected HMECs. The expression of genes that are involved in signal transduction, cell cycle, cell survival, adhesion, angiogenesis, DNA repair, were examined by the RT^2^ Profiler PCR arrays (**Supplementary Tables 2**, **3**).

**Figure 2:**
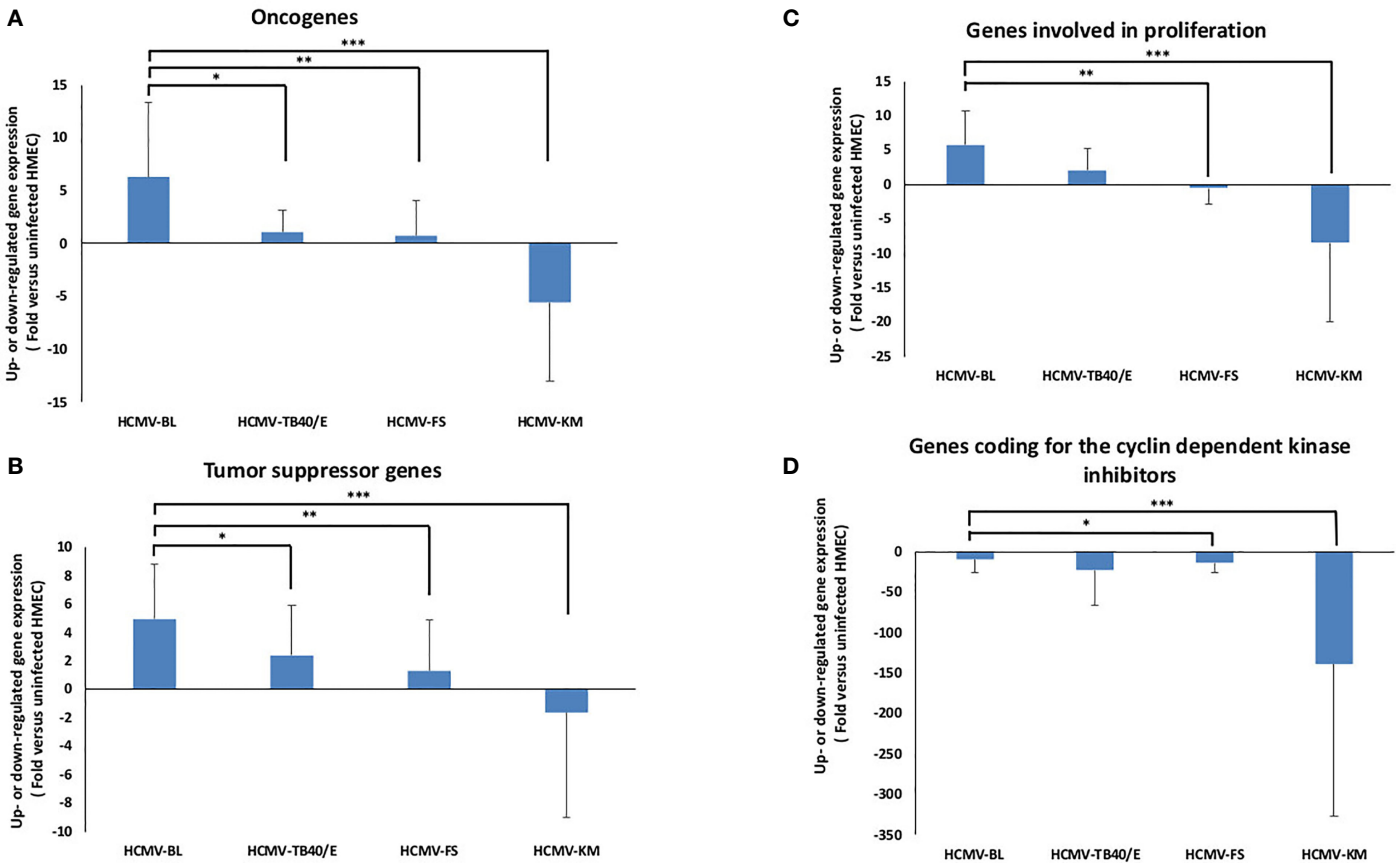
Bulk analysis of the transcriptome differences in oncogenes, tumor suppressor genes, genes involved in cell proliferation and genes coding for cyclin dependent kinase inhibitors in HMECs infected with low-risk and high-risk HCMV strains. **(A)** Assessment of the bulk oncogene expression (based on MYC, FOS, KRAS, JUN, and E2F1 analysis). **(B)** Assessment of the bulk expression of tumor suppressor genes (based on TP53, TP73, SMAD4, VHL, RB, TSC1, and MDM2 analysis). **(C)** Assessment of the bulk expression of genes involved in cell proliferation (based on STAT3, CCND1, and MKI67 analysis). **(D)** Assessment of the bulk expression of genes coding for the cyclin-dependent kinase inhibitors (based on CDKN1A, CDKN2A, CDKN2B, and CDKN3 analysis). The gene expression was measured in HMECs infected with HCMV-BL, HCMV-TB40/E, HCMV-FS and HCMV-KM (MOI = 1) and compared to uninfected HMECs. Histograms represent mean ± SD of three independent experiments. *p-values* were determined by Wilcoxon test. *p-value < 0.05; **p-value < 0.01; ***p-value < 0.001.

**Figure 3:**
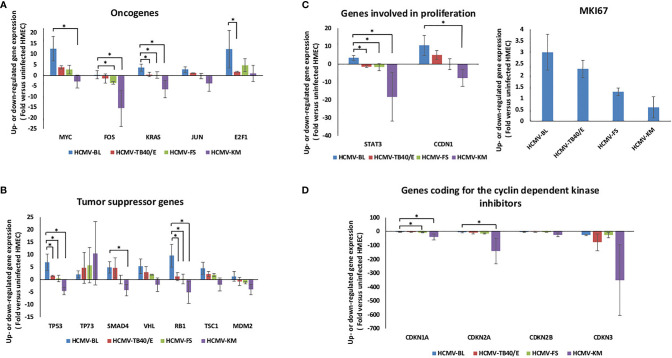
Detailed transcriptome differences in oncogenes, tumor suppressor genes, genes involved in cell proliferation, and genes coding for the cyclin dependent kinase inhibitors in HMECs infected with low-risk and high-risk HCMV strains. **(A)** Assessment of the individual oncogenes expression including MYC, FOS, KRAS, JUN, and E2F1. **(B)** Assessment of the individual expression of tumor suppressor genes: TP53, TP73, SMAD4, VHL, RB, TSC1, and MDM2. **(C)** Assessment of the individual expression of genes involved in cell proliferation such as STAT3, CCDN1, and MKI67. **(D)** Assessment of the individual expression of genes coding for the cyclin-dependent kinase inhibitors: CDKN1A, CDKN2A, CDKN2B, and CDKN3. The gene expression was measured in HMECs infected with HCMV-BL, HCMV-TB40/E, HCMV-FS and HCMV-KM (MOI = 1) and compared to uninfected HMECs. Results represent mean ± SD of three independent experiments. *p-values* were determined by Wilcoxon test. *p-value < 0.05.

The individual gene expression of the oncogenes *Myc* (MYC), *Fos* (FOS), *Jun* (JUN), *KRas* (KRAS), and *E2F1* was assessed in HMECs infected with the five strains HCMV-BL, TB40/E, FS, KM and SC compared to uninfected HMECs (**Figures 2A**, **3A** and **Supplementary Figure 2C**). Values for individual genes were pooled to constitute the overall expression of the oncogenes group **(** **Figure 2A** **)**. In HMECs infected with HCMV-BL, the expression of the oncogenes group was 5.8-fold higher compared to HMECs infected with the TB40/E strain (*p-value*_(HCMV-BL: HCMV-TB40/E)_ = 0.02), 8.5-fold higher compared to HMECs infected with FS strain (*p-value*_(HCMV-BL: HCMV-FS)_ = 0.004) (**Figure 2A**). However, the expression of these oncogenes was downregulated in HMECs infected with the KM strain (*p-value*_(HCMV-BL: HCMV-KM)_ = 0.0001) (**Figure 2A**). Among the oncogenes, Myc and E2F1 were mostly increased in HMECs infected with HCMV-BL compared to HCMV-TB40/E (*p-value*_(Myc- HCMV-BL: HCMV-TB40/E)_ = 0.12; *p-value*_(E2F1- HCMV-BL: HCMV-TB40/E)_= 0.04), HCMV-FS (*p-value*_(Myc- HCMV-BL: HCMV-FS)_ = 0.12; *p-value*_(E2F1- HCMV-BL: HCMV-FS)_ = 0.27) and HCMV-KM (*p-value*_(Myc- HCMV-BL: HCMV-KM)_ = 0.04; *p-value*_(E2F1- HCMV-BL: HCMV-KM)_ = 0.12) infected HMECs (**Figure 3A**). Furthermore, the expression of other oncogenes was tested for HMECs infected with the high-risk strain BL and the low-risk strain FS versus uninfected HMECs. We observed an upregulation of the transcripts of numerous other oncogenes such as KITLG, MYB, RARA, ROS1, and RUNX1 in HMECs infected with HCMV-BL and HCMV-FS (**Table 1**). The upregulation of KITLG, RARA, and ROS1 genes expression was at least 2-fold higher in HMECs infected with HCMV-BL compared to HMECs infected with HCMV-FS **(** **Table 1** **).** In HMECs infected with HCMV-SC, the oncogenes’ expression (MYC, FOS, KRAS, JUN, E2F1) was low, likewise in HMECs infected with HCMV-FS (**Supplementary Figure 2C**).

**Table 1:** The fold change of several oncogenes’ expression in HMECs infected with HCMV-BL and HCMV-FS versus uninfected HMECs.

Gene expression in HMECs infected with(Fold versus uninfected HMECs)		
Gene	HCMV-BL	HCMV-FS
**KITLG**	5.04*	1.93
**MYB**	11.10	6.15
**RARA**	74.67*	36.28
**ROS1**	29.50*	14.04
**RUNX1**	3.49	2.45

*Upregulated gene expression at least two times more in HMEC infected with HCMV-BL compared to HCMV-FS.

The individual gene expression of the tumor suppressor genes coding for p53 protein (TP53), retinoblastoma protein (Rb), p73 protein (TP73), SMAD4, VHL, TSC1, and MDM2 was also assessed in the previously mentioned five strains compared to uninfected HMECs (**Figures 2B**, **3B** and **Supplementary Figure 2C**). Values for individual genes were pooled to constitute the overall expression of the tumor suppressor genes group (**Figure 2B**). The upregulation of tumor suppressor genes’ expression was higher in HMECs infected with the BL strain compared to HMECs infected with the TB40/E and FS by 2 (*p-value*_(HCMV-BL: HCMV-TB40/E)_ = 0.02) and 3.8 fold (*p-value*_(HCMV-BL: HCMV-FS)_ = 0.002) respectively (**Figures 2B**). Nevertheless, the expression of these tumor suppressor genes was downregulated in HMECs infected with HCMV-KM (*p-value*_(HCMV-BL: HCMV-KM)_ = 0.00001) (**Figure 2B**). Among the tumor suppressor genes, TP53, VHL, Rb, and TSC1 expression was mainly increased in HMECs infected with HCMV-BL compared to HCMV-TB40/E (*p-value*_(TP53- HCMV-BL: HCMV-TB40/E)_ = 0.04; *p-value*_(SMAD4- HCMV-BL: HCMV-TB40/E)_ = 0.82; *p-value*_(VHL- HCMV-BL: HCMV-TB40/E)_ = 0.27; *p-value*_(Rb- HCMV-BL: HCMV-TB40/E)_ = 0.04; *p-value*_(TSC1- HCMV-BL: HCMV-TB40/E)_ =0.51), HCMV-FS (*p-value*_(TP53- HCMV-BL: HCMV-FS)_ = 0.04; *p-value*_(SMAD4- HCMV-BL: HCMV-FS)_ = 0.12; *p-value*_(VHL- HCMV-BL: HCMV-FS)_ = 0.51; *p-value*_(Rb- HCMV-BL: HCMV-FS)_= 0.04; *p-value*_(TSC1- HCMV-BL: HCMV-FS)_ =0.51), and HCMV-KM (*p-value*_(TP53- HCMV-BL: HCMV-KM)_ = 0.04; *p-value*_(SMAD4- HCMV-BL: HCMV-KM)_= 0.04; *p-value*_(VHL- HCMV-BL: HCMV-KM)_ = 0.12; *p-value*_(Rb- HCMV-BL: HCMV-KM)_= 0.04; *p-value*_(TSC1- HCMV-BL: HCMV-KM)_ =0.12) (**Figure 3B**). In HMECs infected with HCMV-SC, the tumor suppressor genes (TP53, VHL, TSC1) were slightly expressed, similarly in HMECs infected with HCMV-FS (**Supplementary Figure 2C**). The expression of TP73, a tumor suppressor gene, was markedly increased in HMECs infected with low-risk HCMV strains (KM and SC) and to a lower extent with FS and TB40/E strains compared to HMECs infected with high-risk BL strain (**Figure 3B** and **Supplementary Figure 2C**).

We then analyzed the individual expression of genes involved in cell proliferation namely STAT3, cyclin D1 (CCND1), and Ki67 antigen (MKI67). Interestingly, the STAT3/Cyclin D1 axis is activated in several cancer types including breast cancer (34). Values for individual genes were pooled to constitute the overall expression of the genes involved in cell proliferation group (**Figure 2C**). The expression of “proliferation” genes all together was 2.81 times more upregulated in the HMECs infected with the BL strain compared to HMECs infected with the TB40/E strain (*p-value*_(HCMV-BL: HCMV-TB40/E)_ = 0.05) (**Figure 2C**). While the expression of “proliferation” genes was almost unchanged in HMECs infected with HCMV-FS compared to uninfected HMECs (*p-value*_(HCMV-BL: HCMV-FS)_ = 0.005), it was downregulated in HMECs infected with HCMV-KM (*p-value*_(HCMV-BL: HCMV-KM)_ = 0.0007) (**Figure 2C**). The *STAT3* gene expression was up-regulated in HMECs infected with HCMV-BL in contrast to HMECs infected with HCMV-TB40/E (*p-value*_(HCMV-BL: HCMV-TB40/E)_ = 0.04) and HCMV-FS (*p-value*_(HCMV-BL: HCMV-FS)_ = 0.04) noting that it was substantially down-regulated in HMECs infected with HCMV-KM (*p-value*_(HCMV-BL: HCMV-KM)_ = 0.04) (**Figure 3C**). The gene expression of cyclin D1 was up-regulated in HMECs infected with HCMV-BL compared to uninfected controls. In HMECs infected with HCMV-BL, cyclin D1 gene expression was 2 times more increased when compared to HMECs infected with HCMV-TB40/E (*p-value*_(HCMV-BL: HCMV-TB40/E)_ = 0.12). In contrast, the expression of cyclin D1 was almost unchanged in HMECs infected with HCMV- FS compared to uninfected HMECs (*p-value*_(HCMV-BL: HCMV-FS)_ = 0.12), and down-regulated with in HMECs infected with HCMV-KM (*p-value*_(HCMV-BL: HCMV-KM)_ = 0.04) (**Figure 3C**). In agreement with the highest expression of cyclin D1 in HMECs infected with HCMV-BL and the lowest expression in HMECs infected with HCMV-KM strain, we observed the highest expression of the proliferation marker Ki67 gene (MKI67) in the former and very low expression in the latter (3-fold versus 0.6-fold increase compared to uninfected cells) (**Figure 3C**). HMECs infected with HCMV-SC showed a low expression of the genes involved in proliferation (CCDN1, MKI67) likewise in HMECs infected with the low-risk strains (**Supplementary Figure 2C**). We also assessed the expression of CDK inhibitors CDKN1A, CDKN2A, CDKN2B and CDKN3. Values for individual genes were pooled to constitute the overall expression of CDK inhibitors group (**Figure 2D**). The expression of CDK inhibitors genes was slightly decreased in HMECs infected with HCMV-BL compared to the other three strains TB40/E (*p-value*_(HCMV-BL: HCMV-TB40/E)_ =0.81), FS (*p-value*_(HCMV-BL: HCMV-FS)_ =0.02) and KM (*p-value*_(HCMV-BL: HCMV-KM)_ = 0.001) (**Figures 2D**, **3D**). HMECs infected with HCMV-SC showed a minimal expression of the genes coding for the cyclin dependent kinase inhibitors (**Supplementary Figure 2C**).

### HMECs Infected With Low-Risk and High-Risk HCMV Clinical Strains Display Distinct Expression of Cell Survival, Cell Adhesion, Angiogenesis, and EMT Genes

**Figure 4:**
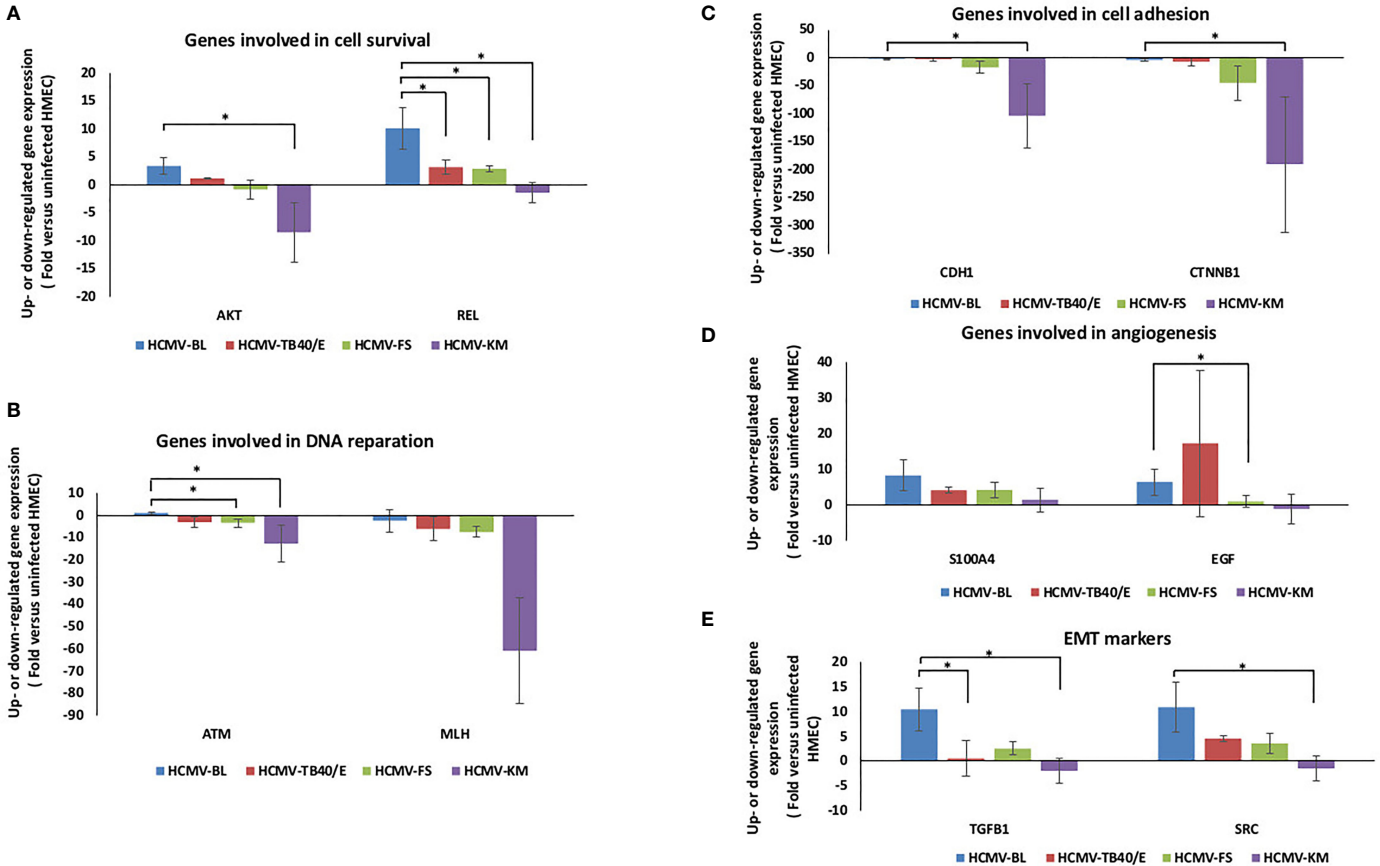
Transcriptome differences in genes involved in cell survival, DNA reparation, cell adhesion, angiogenesis, and EMT markers in HMECs infected with low-risk and high-risk HCMV strains. Up- and downregulation of the expression of genes involved in **(A)** cell survival such as AKT and REL, **(B)** in DNA reparation (ATM and MLH), **(C)** in cell adhesion such as E-cadherin (CDH1) and beta-catenin (CTNNB1), **(D)** in angiogenesis (S100A4 and EGF) and **(E)** of the EMT markers (TGFB1 and SRC). The gene expression was measured in HMECs infected with HCMV-BL, HCMV-TB40/E, HCMV-FS and HCMV-KM (MOI = 1) and compared to uninfected HMECs. Data are represented as mean ± SD of three independent experiments. *p-values* were determined by Wilcoxon test. *p-value < 0.05.

The expression of genes involved in cell survival, such as *AKT*, was upregulated in HMECs infected with BL, and TB40/E strains unlike in HMECs infected with FS and KM strains (*p-value*_(HCMV-BL: HCMV-FS)_ = 0.12 and *p-value*_(HCMV-BL: HCMV-KM)_ =0.04) (**Figure 4A**). In addition, REL was upregulated more in HCMV-BL than in HCMV-TB40/E (*p-value*_(HCMV-BL: HCMV-TB40/E)_ =0.04) and HCMV-FS (*p-value*_(HCMV-BL: HCMV-FS)_=0.04) and was downregulated in HCMV-KM (*p-value*_(HCMV-BL: HCMV-KM)_ =0.04) (**Figure 4A** **)**. The expression of genes involved in DNA reparation (*ATM and MLH*) was downregulated in HMECs infected with HCMV-TB40/E, FS and KM compared to HMEC infected with HCMV-BL (*p-value*_(ATM-HCMV-BL: HCMV-KM)_ =0.04 and *p-value*_(ATM-HCMV-BL: HCMV-FS)_ =0.04) (**Figure 4B**). The gene expression of the molecules involved in cell adhesion such as E-cadherin (CDH1) and beta-catenin (CTNNB1) was minimal in HMECs infected with the four strains (BL, TB40/E, FS and KM) noting that it was mostly downregulated in HCMV-KM (*p-value*_(CDH1-HCMV-BL: HCMV-KM)_ =0.04 and (*p-value*_(CTNNB1-HCMV-BL: HCMV-KM)_ =0.04) (**Figure 4C**). The genes involved in angiogenesis regulation *S100A4 and EGF* were highly expressed in HMECs infected with HCMV-BL compared to HMECs infected with HCMV-FS and HCMV-KM (*p-value*_(S100A4-HCMV-BL: HCMV-KM)_ =0.1; *p-value*_(S100A4-HCMV-BL: HCMV-FS)_ =0.5; *p-value*_(S100A4-HCMV-BL: HCMV-TB40/E)_ =0.1; *p-value*_(EGF-HCMV-BL: HCMV-KM)_ =0.2; *p-value*_(EGF-HCMV-BL: HCMV-FS)_ =0.05 *p-value*_(EGF-HCMV-BL: HCMV-TB40/E)_ =0.5) (**Figure 4D**). Furthermore, the expression of genes involved in proteolysis including matrix metallopeptidase 9 (MMP9) and cathepsin D (CTSD) was 7-fold and 2.4-fold higher respectively, in HMECs infected with HCMV-BL compared to HMECs infected with HCMV-FS (**Supplementary Table 4**). The expression of the EMT markers for instance the transforming growth factor-beta 1 (TGFB1) and the proto-oncogene tyrosine-protein kinase (SRC) was enhanced in HMECs infected with HCMV-BL compared to cells infected with HCMV-TB40/E (*p-value*_(TGFB1- HCMV-BL: HCMV-TB40/E)_ = 0.04; *p-value*_(SRC- HCMV-BL: HCMV-TB40/E)_ = 0.27), FS (*p-value*_(TGFB1- HCMV-BL: HCMV-FS)_ = 0.12; *p-value*_(SRC- HCMV-BL: HCMV-FS)_= 0.12) and KM (*p-value*_(TGFB1- HCMV-BL: HCMV-KM)_ = 0.04; *p-value*_(SRC- HCMV-BL: HCMV-KM)_= 0.04) (**Figure 4E**). In HMECs infected with HCMV-SC, the genes involved in cell survival (AKT, REL), DNA reparation (MLH), and angiogenesis (S100A4) as well as the EMT marker (SRC) were regulated in a similar manner compared to HMECs infected with the FS strain (**Supplementary Figure 2C**).

### Mammosphere Formation in HMECs Infected With Low-risk and High-Risk HCMV Clinical Strains

**Figure 5:**
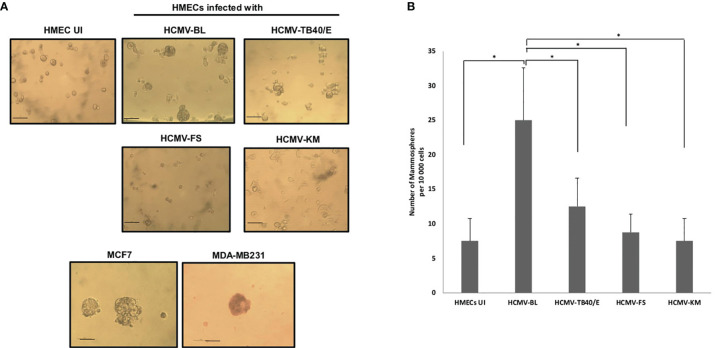
Mammosphere formation in HMEC cultures infected with low-risk (HCMV-TB40/E, FS, and KM) and high-risk (HCMV-BL) strains. **(A)** Mammospheres were observed under an inverted light microscope (Magnification ×100, scale bare 100µm). MCF7 and MDA-MB231 cells were used as positive controls. Uninfected HMECs were used as a negative control. **(B)** The histogram represents the number of mammospheres per 10 000 cells in uninfected and infected HMECs. Data are represented as mean ± SD of three independent experiments. *p-values* were determined by Wilcoxon test. *p-value < 0.05.

We previously observed that the high-risk strains (HCMV-BL and DB) favor the appearance of stemness in chronically infected HMECs and CMV-transformed HMECs (CTH cells) (21), further suggesting that HCMV strains that trigger cellular transformation could also promote stemness. Therefore, HMECs were infected with the high-risk strain HCMV-BL, the low-risk strains HCMV-FS and HCMV-KM, and the prototypic HCMV-TB40/E. At day 1 post-infection, mammosphere formation assay was performed as previously described (35, 36). When we challenged these HCMV-infected cultures to form mammospheres, we detected numerous mammospheres in HCMV-BL infected HMECs as previously reported for HCMV-DB infected cells (*p-value*_(HCMV-BL: HMEC UI)_ =0.03) (37), whereas HCMV-TB40/E displayed a limited number of mammospheres (*p-value*_(HCMV-TB40/E: HMEC UI)_ = 0.07); only few mammospheres were observed in HMECs infected with HCMV-FS and HCMV-KM (**Figure 5**). The breast cancer cell lines MCF7 and MDA-MB231 generated mammospheres as previously reported (37) (**Figure 5**). Moreover, few mammospheres were observed in uninfected HMECs as previously described (38) (**Figure 5**). Altogether, we noticed a preferential formation of mammospheres in HMECs infected with the high-risk HCMV-BL strain in opposition to the low-risk HCMV-TB40E (*p-value*_(HCMV-BL: HCMV-TB40/E)_ = 0.03), HCMV-FS (*p-value*_(HCMV-BL: HCMV-FS)_ = 0.03) and HCMV-KM (*p-value*_(HCMV-BL: HCMV-KM)_ = 0.03) strains.

### Side-By-Side Comparison of the Prototypic Strain TB40/E and the High-Risk DB Strain for Some Specific Phenotypic and Functional Traits

**Figure 6:**
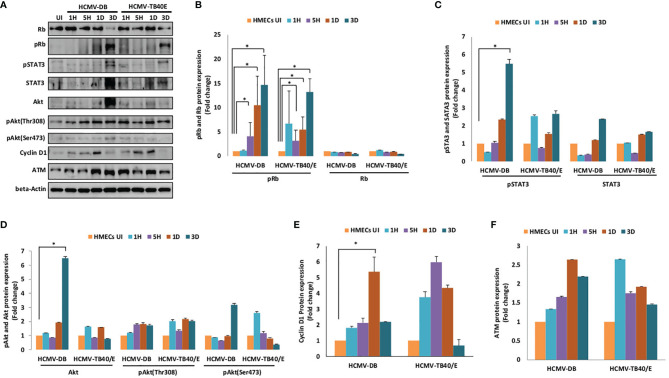
Distinct activation of oncogenic pathways in HMECs infected with the high-risk DB strain and the prototypic TB40/E strain. **(A)** Immunoblotting data of Rb, pRb, STAT3, pSTAT3, Akt, pAkt-Thr308, pAkt-Ser473, cyclin D1 and ATM in uninfected HMECs and HMECs infected with HCMV-DB and HCMV-TB40/E (MOI = 1) at 1H, 5H, D1 and D3 post-infection. β-actin was used as loading control. Results are representative of three independent experiments. Histograms representing **(B)** Rb, pRb, **(C)** STAT3, pSTAT3 **(D)** Akt, pAkt-Thr308, pAkt-Ser473, **(E)** cyclin D1, and **(F)** ATM expression in HMECs infected with HCMV-DB and HCMV-TB40/E (MOI = 1) at 1H, 5H, D1 and D3 post-infection; protein expression is shown based on quantification by ImageJ 1.40 software. Data are represented as mean ± SD of three independent experiments. *p-values* were determined by Wilcoxon test. *p-value < 0.05.

Since we observed limited mammospheres in HMECs infected with HCMV-TB40/E (**Figure 5**) and a slight increase in telomerase activity which has been previously reported (20), we decided to compare side by side the activation of oncogenic molecular pathways in HMECs infected with HCMV-TB40/E and the high-risk HCMV-DB strain. The productive HCMV-DB and TB40/E infection was confirmed in HMECs where both confocal microscopy imaging and flow cytometry data showed the IE1 staining **(** **Figures 1A, B** **)**. At the protein expression level (day 3 post-infection), Rb was diminished whereas pRb was highly expressed in HMEC-HCMV-DB (*p-value*_(HCMV-DB: HMEC-UI)_ = 0.04) and to a lesser extent in HMEC-HCMV-TB40/E (*p-value*_(HCMV-TB40/E: HMEC-UI)_ = 0.04, *p-value*_(HCMV-TB40/E: HCMV-DB)_ = 0.8) (**Figures 6A, B**). At the same time point, pSTAT3 expression was higher in HMEC-HCMV-DB (*p-value*_(HCMV-DB: HMEC-UI)_ = 0.03) compared to HMEC-HCMV-TB40/E (*p-value*_(HCMV-TB40/E: HMEC-UI)_ = 0.06) and STAT3 expression was further increased in HMEC-HCMV-DB compared to HMEC-HCMV-TB40/E (**Figures 6A, C**). Further, Akt expression was highly increased in HMEC-HCMV-DB (*p-value*_(HCMV-DB: HMEC-UI)_ = 0.03), compared to HMEC-HCMV-TB40/E at day 3 post-infection (*p-value*_(HCMV-TB40/E: HMEC-UI)_ = 0.06), and an increase in its phosphorylation on both serine 473 and threonine 308 in HMEC-HCMV-DB was observed (**Figures 6A, D**). At day 1 post-infection, cyclin D1 expression was increased in HEMC-HCMV-DB (*p-value*_(HCMV-DB: HMEC-UI)_ = 0.03) compared to HMEC-HCMV-TB40/E (*p-value*_(HCMV-TB40/E: HMEC-UI)_ = 0.06) (**Figures 6A, E**). The protein expression of ATM was elevated in HMECs infected with HCMV-DB in comparison to HCMV-TB40/E (**Figures 6A, F**). Our results indicate that the HCMV-TB40/E strain does not induce significant activation of the molecular oncogenic pathways, especially when compared to the high-risk strain HCMV-DB, and therefore should be classified as a low-risk HCMV strain consistent with the absence of colony formation in soft agar in HMEC cultures seeded with TB40/E.

### Detection of HCMV in Breast Cancer Biopsies

**Figure 7:**
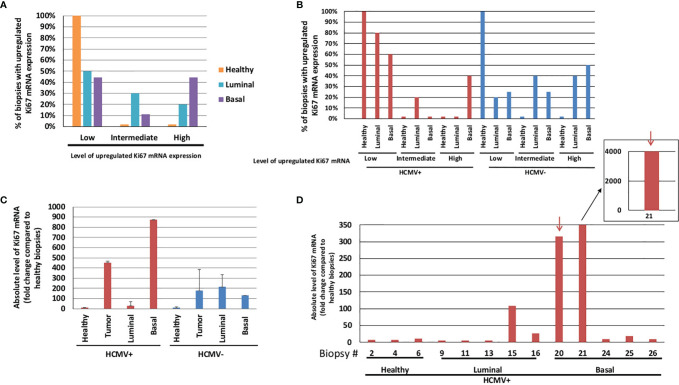
Ki67 gene expression parallel to HCMV detection in healthy and tumor (luminal and basal) breast biopsies. **(A)** Histograms representing the percentage of healthy and tumor (luminal and basal) biopsies with an upregulated Ki67 mRNA expression compared to healthy tissue **(B)** in the presence/absence of HCMV. (Level of upregulated Ki67 mRNA expression: Low<30, 30<Intermediate <100, and High >100) **(C)** Histogram showing the mean ± SD of absolute levels of Ki67 mRNA in healthy, tumor (luminal + basal), luminal, and basal biopsies which were grouped into HCMV-positive and HCMV-negative biopsies. **(D)** Detailed absolute levels of Ki67 mRNA in HCMV-positive biopsies (healthy, luminal, and basal biopsies). *Red arrows*, two HCMV strains detected in basal tumors which expressed high Ki67 levels. Each number on the X-axis represents one healthy, luminal or basal biopsy. Biopsy details can be found in **Supplementary Table 1** and in *Materials and Methods*.

To assess the impact of HCMV strains on cellular gene expression *in vivo*, Ki67 expression was quantified by RT-qPCR in breast tumor biopsies, including luminal and basal-like tumors compared to healthy breast tissues. First, we measured the percentage of healthy and tumor biopsies with low (<30), intermediate (30–100) or high (>100) Ki67 mRNA expression (**Figures 7A, B**). Basal-like biopsies accounted for the highest percentage of biopsies with high Ki67 mRNA expression level (% of basal-like: 44%) compared to healthy (2%) and luminal biopsies (20%) (*p-value*_(High Ki67 - Basal : Healthy)_ = 0.02) (**Figure 7A**). To further study the role of HCMV associated with Ki67 transcript expression, we assessed the presence of HCMV in the biopsies by quantifying *IE1* and *UL69* genes using qPCR (21, 30). In HCMV-positive biopsies, basal-like biopsies showed the highest percentage of biopsies with high levels of Ki67 mRNA (40%) in contrast to the healthy (0%) and luminal (0%) biopsies (**Figure 7B**). Second, we assessed the absolute level of Ki67 mRNA per biopsy of healthy tissues, luminal and basal-like tumors taking into account the presence or absence of HCMV (**Figure 7C**). Healthy biopsies showed low absolute levels of Ki67 mRNA compared to tumor biopsies independent of HCMV status (presence or absence) (*p-value* = 0.02). In tumor biopsies, the absolute level of Ki67 mRNA was enhanced by 2.5-fold in HCMV-positive biopsies compared to HCMV-negative biopsies (*p-value* = 0.2). In HCMV-positive biopsies, the absolute level of Ki67 mRNA in basal tumors was enhanced by 31.45-fold compared to luminal tumors (*p-value* = 0.17) **(** **Figure 7C**). The absolute level of Ki67 mRNA was shown separately for each biopsy where cutoff lines were set to differentiate between low and high Ki67 expression and allow a closer look on the variability of Ki67 mRNA expression per sample **(** **Supplementary Figure 3** **)**. Thus, when we independently measured the absolute level of Ki67 mRNA in each HCMV-positive biopsy, we detected two basal biopsies (biopsies #20 and #21) showing the highest absolute levels of Ki67 mRNA with 315 and 4015-fold increase versus the mean value of healthy tissue, respectively (**Figure 7D**). Meanwhile, much lower absolute levels of Ki67 mRNA were detected in the other HCMV-positive tumor and healthy biopsies. Low absolute levels of Ki67 mRNA were detected in HCMV-positive luminal biopsies (less than 30-fold increase versus mean value of healthy tissue) except for one biopsy (biopsy #15 with 108-fold increase) (**Figure 7D**).

Within our data, we were able to identify two biopsies, namely biopsies #20 and #21 (**Figure 7D**, arrows), that could harbor high-risk HCMV strains. Given the total number of biopsies, we might not be able to properly identify all the high-risk strains, which effect might be partially masked in the overall HCMV-positive biopsies. To confirm the high-risk status of biopsies #20 and #21, we isolated the strains for each biopsy and used it to infect HMECs (**Supplementary Figure 4A**). After few weeks in culture, we observed dense structures (**Supplementary Figure 4B**), similar to the morphology of the previously reported CTH cells (20, 21). These emerging structures were not present in HMECs infected with low-risk strains that were isolated from other biopsies in which we observed cell lysis. Overall, we identified two high-risk strains (n=2; 15%) out of the HCMV-positive biopsies (n=13); upon assessing the proportion of high-risk strain within tumor biopsies only, we identified two high-risk strains (n=2; 20%) out of the total HCMV-positive tumors (n=10).

Finally, using twenty different breast tumor biopsies, we aimed at evaluating the value of Ki67 expression as an index for increased proliferation in breast cancer. Ki67 mRNA and protein levels were measured by RT-qPCR and IHC, respectively, on the same biopsy sample. A correlation was observed between Ki67 protein and mRNA levels (**Supplementary Figure 5**). This data indicates that Ki67 mRNA expression could be considered as a reliable marker for the measurement of increased proliferation in breast cancer.

## Discussion

**Figure 8:**
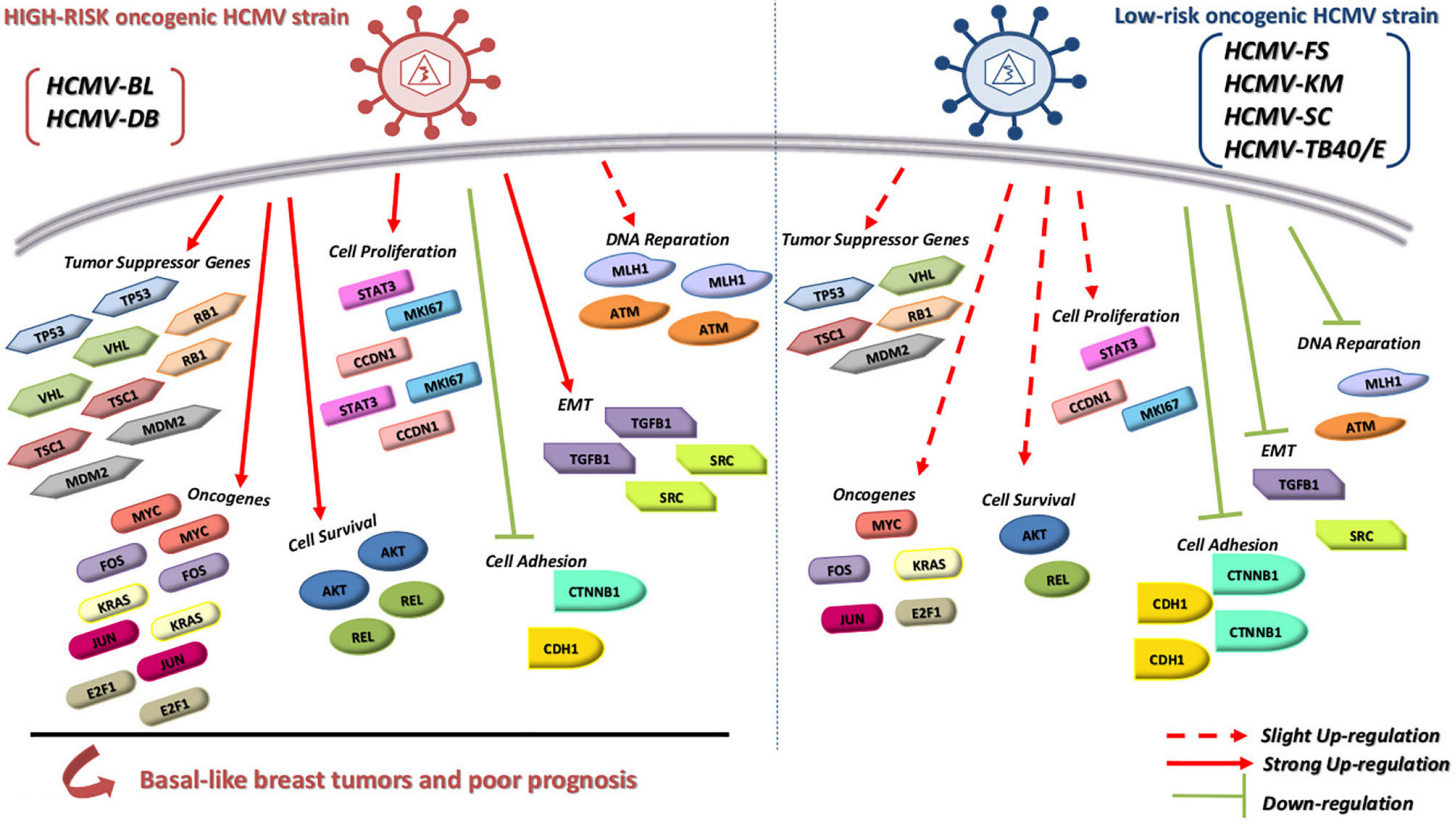
A hypothetical model to summarize the transcriptome differences between low-risk and high-risk HCMV strains based on the modulation of cellular pathways involved in oncogenesis. In contrast to the low-risk HCMV strains (e.g. HCMV-FS, HCMV-KM and HCMV-TB40/E), the high-risk HCMV strains (e.g. HCMV-DB and HCMV-BL) strongly upregulate the expression of oncogenes, cell proliferation genes, genes involved in cell survival and EMT genes, thereby they might favor the initiation and/or the progression of basal breast tumors with poor prognosis.

We previously isolated the two clinical isolates, HCMV-DB and HCMV-BL, which in primary human mammary epithelial cells (HMECs) promoted oncogenic molecular pathways, established anchorage-independent growth *in vitro*, and produced tumorigenicity in mice models (20, 21), therefore named high-risk oncogenic strains. In contrast, other clinical HCMV strains such as HCMV-FS, KM and SC did not trigger such traits (21), therefore named low-risk oncogenic strains. TB40/E strain did not induce significant activation of the molecular oncogenic pathways in HMECs, especially when compared to the high-risk strain HCMV-DB, and therefore should be classified as a low-risk HCMV strain consistent with the absence of colony formation in soft agar. In contrast to the other HCMV low-risk strains, HCMV-KM was isolated from a two-year old infant with HCMV congenital infection which might explain the different observed transcriptome profile compared to other low-risk HCMV strains (39). Our results indicate the capacity of the six tested HCMV clinical strains to replicate efficiently in HMEC cells allowing the comparison of their transcriptome profile. The transcriptome of HMECs infected with HCMV-BL strain exhibits a pro-oncogenic cellular environment with enhanced expression of several oncogenes, proliferation markers, cell survival genes, and EMT traits **(** **Figure 8** **)**. Moreover, these pro-oncogenic characteristics were accompanied with stemness traits as assessed *in vitro* by mammosphere formation. In contrast, based on the transcriptome analysis, the HCMV-FS, KM and SC strains were less prone to favor an oncogenic environment **(** **Figure 8** **)**. The prototypic TB40/E strain displays mostly low-oncogenic traits as assessed by its proteomic profile in infected HMECs **(** **Figure 8** **)**. Finally, we observed a potential link between the presence of HCMV in the breast tumor, especially the basal tumors, and high levels of Ki67 mRNA further suggesting the presence of high-risk HCMV strains in some of the basal tumors. Altogether, the transcriptome profiles of HCMV-infected HMECs and the analysis of Ki67 mRNA levels in breast tumors might allow for the classification of high and low-risk HCMV strains based on their oncogenic potential.

Although HCMV clinical isolates display a broad cellular tropism, the growth of laboratory HCMV strains is restricted to fibroblasts (16). Furthermore, several clinical HCMV strains can generate distinct inflammatory (M1) and immunosuppressive (M2) macrophage polarization after infection of blood monocytes and tissue macrophage and can establish latency in these latter (17, 23, 25, 40). The preferential tropism of clinical HCMV strains might favor their long-lasting low-level viral replication in HMECs and ultimately promote breast cancer (41–43). Taking into account its tropism for mammary epithelial cells and its transcriptome oncogenic environment, the HCMV-BL strain is close to the previously described HCMV-DB strain (37). Based on their transforming capacity *in vitro* (soft agar), the activation of oncogenic and proliferation pathways including Myc and Ki67 overexpression, and the development of tumors when injected in NSG mice (20, 21) the HCMV-DB and HCMV-BL strains are classified as high-risk strains.

Inactivation of tumor suppressor proteins such as retinoblastoma protein (Rb) and p53 and activation of pro-oncogenes such as Myc and Ras are critical events involved in HMEC cells transformation and immortalization (44, 45) and are known to favor breast cancer progression (46). Intriguingly, the expression of p53 was 7-fold higher in HMECs infected with high-risk strain HCMV-BL compared to uninfected HMECs. Enhanced expression of p53 has been described in human mammary epithelial cells infected with HCMV-DB (20, 37) and in fibroblasts infected with numerous HCMV strains (47, 48). To explain the functional inactivation of p53 needed for cell transformation, a potential inhibitory effect of IE2 on p53 transcriptional activity has been previously reported (49). In addition, we previously described p53 protein binding to IE2 in HCMV-DB infected HMECs, leading to its functional inactivation parallel to p53 transcript upregulation (20, 37). A similar mechanism might be in action in HMECs infected with HCMV-BL.

Likewise, the expression of Rb gene is increased in HMECs infected with HCMV-BL by 10-fold compared to the uninfected HMECs. Several small DNA tumor viruses such as adenovirus, human papillomavirus, and polyomavirus were described to abolish the function of Rb protein (50). In addition, it was reported that Rb inactivation mediated by viral proteins through the E2F transcriptional factor is necessary for viral replication and mammalian cells transformation (50, 51). Also, the inactivation of Rb protein could be caused by post-translation phosphorylation (52). For HCMV, manipulation of the cell cycle through Rb inactivation could favor cell transformation (53). First, the viral protein pp71 degrades the hypophosphorylated Rb protein earlier during infection, then Rb protein reaccumulates later during infection (54–56). Second, the viral protein UL97 phosphorylates Rb protein on several CDK target sites and deactivates it. Third, the viral proteins IE1 and IE2 were described to activate E2F-responsive promoters and most probably induce Rb inactivation (57–59). Interestingly, parallel to enhanced Rb gene expression we observed high levels of E2F1 transcript in HMECs infected with the high-risk BL strain. Thus, the inactivation of both p53 and Rb proteins is mandatory for cell transformation in HCMV-BL and HCMV-DB infected HMECs which might explain that even under enhanced p53 and Rb gene expression, their protein activity will be abolished.

In agreement with our previous results (21), the overexpression of Myc and KRas could participate in the transformation process of HMEC cells infected with HCMV-BL. In addition, the enhanced expression of other oncogenes such as Jun and E2F1 observed in HMECs infected with HCMV-BL could stimulate transformation (60, 61). Interestingly, the viral proteins IE1 and IE2 induce Myc activation (62). We observed previously a similar activation of Myc and Ras protein and gene expression in HMECs infected with the HCMV-DB strain (20, 37).

Furthermore, one of the mechanisms described to transform HMECs is the link of Myc overexpression to PI3K/Akt pathway activation (63, 64). We observed mostly enhanced expression of Akt gene in HMECs infected with HCMV-BL compared to HCMV-FS, KM and TB40/E in agreement with our previous results (21), and an upregulation of serine-473 phosphorylation of Akt in HMECs infected with HCMV-DB compared to HCMV-TB40/E. The PI3K/Akt activation in HCMV infected cells is in line with previous findings (65). In addition, several studies have pointed the role of REL in cell growth and oncogenesis (66). The overexpression of REL was associated with cell transformation *in vitro* and/or *in vivo.* Epstein-Barr virus (EBV) activates REL/NF-kB during malignant transformation (67). Thus, the overexpression of REL in HMECs infected with HCMV-BL is consistent with its high oncogenic potential.

In agreement with the HCMV-DB transcriptome study (37), an enhanced expression of proliferation markers such as STAT3, cyclin D1, and especially Ki67 was observed in HMEC cells infected with HCMV-BL but not with HCMV- FS, KM, SC and TB40/E. The proliferation and invasion of several cancers could be explained by STAT3 activation and cyclin D1 overexpression (68). Moreover, the overexpression of pSTAT3 and STAT3 at day 3 post-infection was observed mainly in HMECs infected with HCMV-DB, but to a lesser extent with HCMV-TB40/E. These findings confirm that the high-risk HCMV strains such as BL and DB have high proliferation capacities compared to the low-risk HCMV strains, such as TB40/E, FS, KM and SC.

Studies have reported that ataxia telangiectasia mutated (ATM) is required for efficient viral replication of numerous viruses including HCMV, HSV-1, HPV, and HIV (69–73). Indeed, HCMV infection leads to host DNA damage responses (DDRs) with activation of the DNA reparation pathway (74–76). Enhanced expression of ATM gene was observed in infected HMECs with HCMV-BL and with HCMV-DB as reported previously (37). Lower levels of ATM expression were detected in HMECs infected with HCMV-TB40/E, FS, and KM. The enhanced expression of ATM can promote genome instability resulting in cellular transformation. MLH expression is necessary for efficient HSV-1 viral replication (77) and its enhanced expression has been linked to high levels of genomic instability, cancer progression, and aggressiveness (78, 79). Likewise, the overexpression of MMP9 and CTSD observed in HMECs infected with HCMV-BL was associated with the most aggressive subtype of breast cancer (80), tumor cell invasion, and metastasis (81–84).

The expression of genes involved in EMT such as the proto-oncogene tyrosine-protein kinase Src (85) and the transforming growth factor-beta 1 (TGFB1) (86, 87) was higher in HMECs infected with HCMV-BL than with HCMV-TB40/E, FS, KM and SC. Our findings are in accordance with our previous analysis of HCMV-DB transcriptome (37).

Altogether the transcriptome analysis of HMECs infected with the high-risk strain HCMV-BL underlines the potential capacity of the emergence of cancer stem cells (CSCs) compared to the HMECs infected with low-risk strains (HCMV-FS, KM and SC). In line with our transcriptome data and with the previously reported enhanced mammosphere formation in HMECs infected with HCMV-DB (37), we observed that the number of mammospheres detected in HMECs infected with HCMV-BL was 2-fold, 2.8-fold and 3-fold higher compared to HMECs infected with HCMV-TB40/E, HCMV-FS and KM, respectively

The distinct oncogenic potential of HCMV strains observed at the transcriptome level *in vitro* was then assessed *in vivo* in breast tumors. The nuclear protein Ki67 is not required only for cell proliferation in tumor but also for tumor initiation, growth, and metastasis. Moreover, Ki67 inactivation leads to transcriptome remodeling that alters mesenchymal-epithelial balance and suppresses stemness (88). Ki67 is highly expressed in malignant tissues with poorly differentiated tumor cells (89). High Ki67, possessing high proliferation potential, was reported to worsen the survival of triple negative breast cancer patients and to trigger quick tumor recurrence within a short period of time (90). Remarkably, enhanced expression of Ki67 gene and upregulation of Ki67 protein was observed in HMECs infected with the HCMV-DB and BL strains (20, 21). Therefore, we assessed the link between HCMV presence and Ki67 gene overexpression in breast tumor biopsies. Basal-like biopsies revealed the highest percentage of biopsies with high levels of Ki67 mRNA compared to the healthy and luminal biopsies. Only tumor biopsies were impacted by HCMV status; the highest level of Ki67 mRNA was detected in HCMV-positive tumor biopsies compared to HCMV-negative tumor biopsies. Among the HCMV-positive biopsies, the absolute level of Ki67 was low in all healthy and most of the luminal biopsies except for one luminal biopsy (#15 with limited increase in Ki67 absolute level). In contrast, among the tested basal-like HCMV-positive biopsies, we observed augmented absolute levels of Ki67 mRNA in two biopsies (biopsies #20 and #21) despite the low absolute levels of Ki67 mRNA that were detected in some biopsies. The HCMV strains that were isolated from biopsies #20 and 21, transformed the freshly uninfected HMECs resulting in the appearance of CMV-transformed HMECs (CTH cells) *in vitro* indicating their high-risk profile **(** **Supplementary Figure 4** **)**. Since high-risk HCMV strains strongly enhanced the expression of Ki67Ag (20, 21), the majority of the HCMV strains detected in the breast tumor biopsies or in the adjacent tested healthy tissues might be non-oncogenic or low-risk HCMV strains. The detection of very high expression of Ki67 mRNA in two basal tumor biopsies concomitant with the presence of HCMV suggests a potential link between high-risk HCMV strains present in the tumor tissue, highly enhanced Ki67 expression, and the initiation and/or progression of basal breast tumors. In agreement with our results, Ki67 was described as a key marker associated with poor prognosis in triple-negative breast cancer (91). We are currently studying a larger cohort of breast cancer biopsies and assessing additional “oncogenic” markers such as c-Myc to further highlight the exact role of HCMV strains in breast oncogenesis.

## Conclusion

In conclusion, our data indicate the capacity of high-risk HCMV strains to induce a pro-oncogenic cellular environment in contrast to the low-risk strains. Similar to the HCMV-DB strain, HCMV-BL was able to induce enhanced expression of various oncogenes, genes involved in proliferation, cell survival, DNA reparation, and EMT. Additionally, high-risk HCMV strains present stemness traits with enhanced mammosphere formation *in vitro* compared to the low-risk strains. *In vivo*, we detected the concomitant presence of a very high Ki67 gene expression in two HCMV-positive basal breast tumors, indicating a potential link between high-risk HCMV strains present in the tumoral tissue and the development of aggressive tumors of poor prognosis. The discrimination between HCMV clinical strains based on their distinct oncogenic potential will pave the way for understanding their respective role in breast cancer and open the door to new therapeutic approaches.

## Data Availability Statement

The original contributions presented in the study are included in the article/**Supplementary Material**. Further inquiries can be directed to the corresponding author.

## Ethics Statement

The studies involving human participants were reviewed and approved by local ethics committees of Besançon University Hospital (Besançon, France) and the French Research Ministry (AC-2015-2496, CNIL n°1173545, NF-S-138 96900 n°F2015). The patients/participants provided their written informed consent to participate in this study.

## Author Contributions

Conceptualization, GH. Formal analysis, SH, SP, and GH. Investigation, SH, SP, RE, ZN, and CL. Writing—original draft preparation, SH and GH. Writing—review and editing, SH, SP, RE, and GH. Visualization, SH and SP. Supervision, GH. Project administration, GH. Funding acquisition, GH. All authors contributed to the article and approved the submitted version.

## Funding

This work was supported by grants from the University of Franche-Comté (UFC) (CR3300), the Région Franche-Comté (2021-Y-08292 and 2021-Y-08290) and the Ligue contre le Cancer (CR3304) to GH. SH is recipient of a doctoral scholarship from Lebanese municipality. RE is a recipient of a doctoral scholarship from Hariri foundation for sustainable human development.

## Conflict of Interest

The authors declare that the research was conducted in the absence of any commercial or financial relationships that could be construed as a potential conflict of interest.

## Publisher’s Note

All claims expressed in this article are solely those of the authors and do not necessarily represent those of their affiliated organizations, or those of the publisher, the editors and the reviewers. Any product that may be evaluated in this article, or claim that may be made by its manufacturer, is not guaranteed or endorsed by the publisher.
