# Supplementary material for: Distinct Oncogenic Transcriptomes in Human Mammary Epithelial Cells Infected With Cytomegalovirus
Source: Front Immunol. 2021 Dec 22;12:772160. doi: 10.3389/fimmu.2021.772160 (PMC8727587; doi:10.3389/fimmu.2021.772160)
Supplement: Supplementary file 1 [file DataSheet_1.docx]

Supplementary Material

# Supplementary Tables

**Supplementary Table 1. Histopathologic data for luminal and basal breast cancer biopsies.**

|  | **Histologic type** | **Elston-Ellis Grading system** | **ER (%+)** | **PGR (%+)** | **HER2 (%+)** | **Vascular Emboli** | **TNM Staging** |
| --- | --- | --- | --- | --- | --- | --- | --- |
| **LUMINAL (n=10)** | | | | | | | |
| Biopsy N° |  |  |  |  |  |  |  |
| 9 | Lobular | II (3,2,1) | 95 | 10 | 0 | no | T2N0Mx |
| 10 | Ductal | II (3,2,1) | 95 | 90 | 0 | no | T1cN0Mx |
| 11 | Ductal | I (2,1,1) | 100 | 100 | 0 | no | ND |
| 12 | Lobular | II (3,2,1) | 80 | 90 | 0 | no | T2N1Mx |
| 13 | Ductal | I (2,2,1) | 95 | 95 | 0 | no | T1cN1mi |
| 14 | Ductal | II (2,2,3) | 100 | 70 | 0 | ND | T2N1a(sn) |
| 15 | Lobular | II (3,2,1) | 90 | 0 | 0 | no | T2N0Mx |
| 16 | Ductal | II (2,2,3) | 99 | 95 | 0 | ND | T2N0(sn) |
| 17 | Ductal | II (2,3,2) | 90 | 90 | 0 | ND | T2N0(sn) |
| 18 | Ductal | II (3,2,1) | 95 | 50 | 0 | ND | ND |
| **BASAL (n=9)** | | | | | | | |
| 19 | Ductal | III (3,2,3) | 0 | 0 | 0 | ND | ND |
| 20 | Ductal | III (3,3,3) | 0 | 0 | 0 | yes | ND |
| 21 | Ductal | III (3,3,3) | 0 | 0 | 0 | yes | ND |
| 22 | Ductal | III (3,3,3) | 0 | 0 | 0 | ND | T2N0 |
| 23 | Ductal | III (3,3,3) | 0 | 0 | 0 | yes | ND |
| 24 | Ductal | III (3,3,3) | 0 | 0 | 0 | yes | T2N1mi |
| 25 | Ductal | III (3,2,3) | 0 | 0 | 0 | ND | T2N0 |
| 26 | Ductal | III (3,2,3) | 0 | 0 | 0 | no | ND |
| 27 | Ductal | III (3,3,3) | 5 | 0 | 0 | yes | ND |

| ND, not done |
| --- |

**Supplementary Table 2.** **List of tested genes using the Human Breast Cancer RT^2^ profiler PCR assays (PAHS-131ZA).**

| **Gene Symbol** | **Description** |
| --- | --- |
| CTSD | Cathepsin D |
| MKI67 | Antigen identified by monoclonal antibody Ki-67 |
| MMP9 | Matrix metallopeptidase 9 (gelatinase B, 92kDa gelatinase, 92kDa type IV collagenase) |

**Supplementary Table 3.** **List of tested genes using the Oncogenes & Tumor Suppressor genes RT^2^ profiler PCR assays (PAHS-502ZR).**

| **Gene Symbol** | **Description** |
| --- | --- |
| AKT | V-akt murine thymoma viral oncogene homolog 1 |
| ATM | Ataxia telangiectasia mutated |
| CCND1 | Cyclin D1 |
| CDH1 | Cadherin 1, type1 E-cadherin (epithelial) |
| CDKN1A | Cyclin-dependent kinase inhibitor 1A (p21, Cip1) |
| CDKN2A | Cyclin- dependent kinase inhibitor 2A (melanoma, p16, inhibits CDK4) |
| CDKN2B | Cyclin- dependent kinase inhibitor 2B (p15, inhibits CDK4) |
| CDKN3 | Cyclin- dependent kinase inhibitor 3 |
| CTNNB1 | Catenin (cadherin-associated protein), beta1 88kDa |
| E2F1 | E2F transcription factor 1 |
| EGF | Epidermal growth factor |
| FOS | FBJ murine osteosarcoma viral oncogene homolog |
| JUN | Jun proto-oncogene |
| KITLG | KIT ligand |
| KRAS | V-Ki-ras2 Kirsten rat sarcoma viral oncogene homolog |
| MCL1 | Myeloid cell leukemia sequence 1 (BCL2-related) |
| MDM2 | Mdm2 p53 binding protein homolog (mouse) |
| MET | Met proto-oncogene (hepatocyte growth factor receptor) |
| MLH1 | Mult homolog1, colon cancer, nonpolyposis type 2 (E.coli) |
| MYB | V-myb myeloblastosis viral oncogene homolog (avian) |
| MYC | V-myc myeloblastosis viral oncogene homolog (avian) |
| NFKBIA | Nuclear factor of kappa light polypeptide gene enhancer in B-cells inhibitor, alpha |
| PIK3CA | Phosphoinositide-3-kinase, catalytic, alpha polypeptide |
| PML | Promyelocytic leukemia |
| PRKCA | Protein kinase C, alpha |
| RAF1 | V-raf-1 murine leukemia viral oncogene homolog 1 |
| RARA | Retinoic acid receptor, alpha |
| RB1 | Retinoblastoma 1 |
| REL | V-rel reticuloendotheliosis viral oncogene homolog (avian) |
| ROS1 | C-ros oncogene 1, receptor tyrosine kinase |
| RUNX1 | Runt-related transcription factor 1 |
| S100A4 | S100 calcium binding protein A4 |
| SMAD4 | SMAD family member 4 |
| SRC | V-src sacrama (shmidt-ruppin A-2) viral oncogene homolog (avian) |
| STAT3 | Signal transducer and activator of transcription 3 (acute-phase response factor) |
| TGFB1 | Transforming growth factor, beta 1 |
| TP53 | Tumor protein p53 |
| TP73 | Tumor protein p73 |
| TSC1 | Tuberous sclerosis 1 |
| VHL | Von Hippel-Lindau tumor suppressor |

**Supplementary Table 4. Comparison between the expression of some genes involved in proteolysis in HMECs infected with HCMV-BL and HCMV-FS.**

|  | **Up- or down-regulated gene expression in HMECs infected with**  **(Fold versus uninfected HMEC)** | |
| --- | --- | --- |
| **Gene** | **HCMV-BL** | **HCMV-FS** |
| CTSD | 2086.21 | 855.13 |
| MMP9 | 91.56 | 13.64 |

# Supplementary Figures


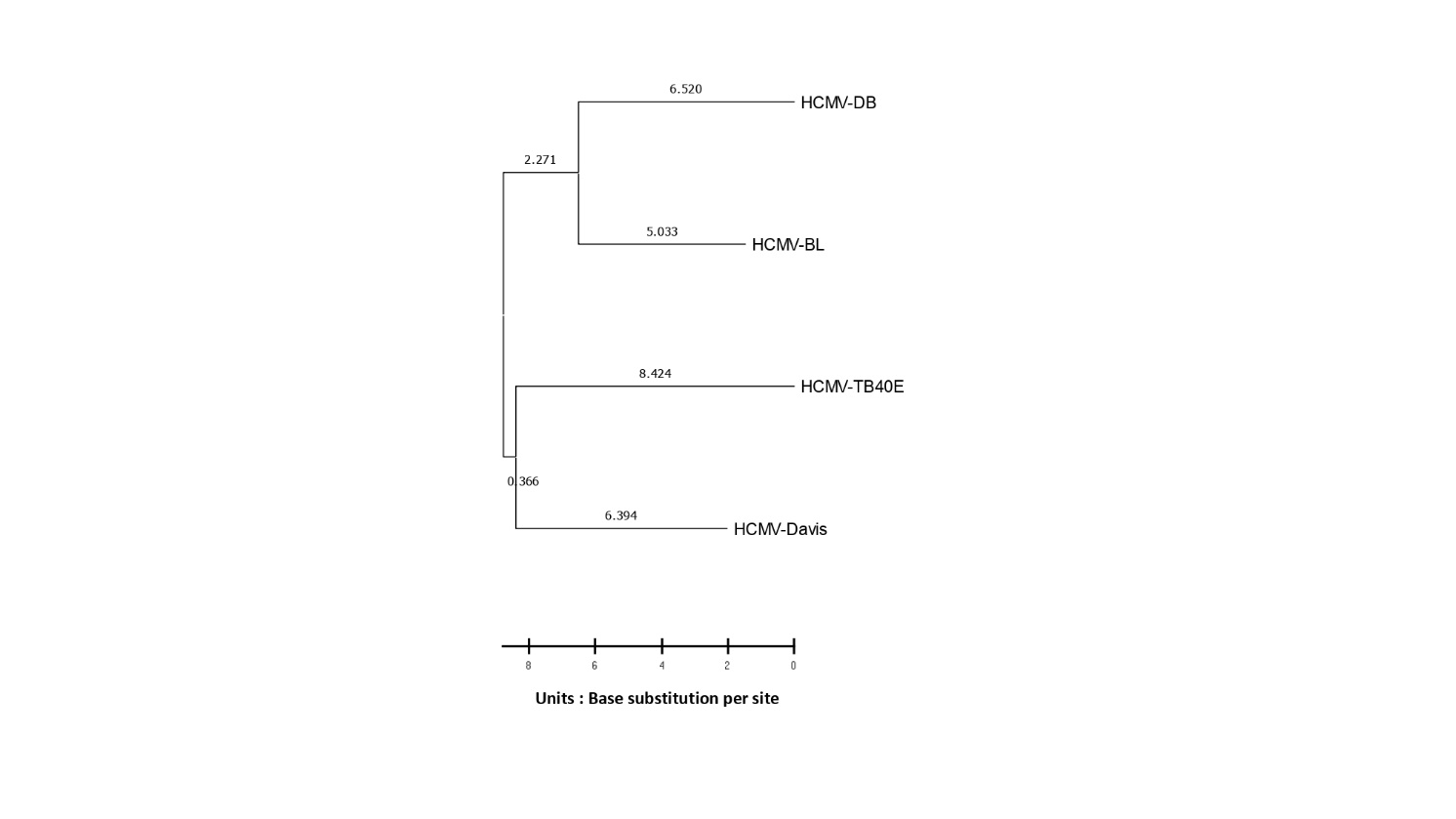


**Supplementary Figure 1.** Phylogenetic analysis comparing the genomic sequences of four HCMV strains (DB, BL, TB40/E, and Davis)


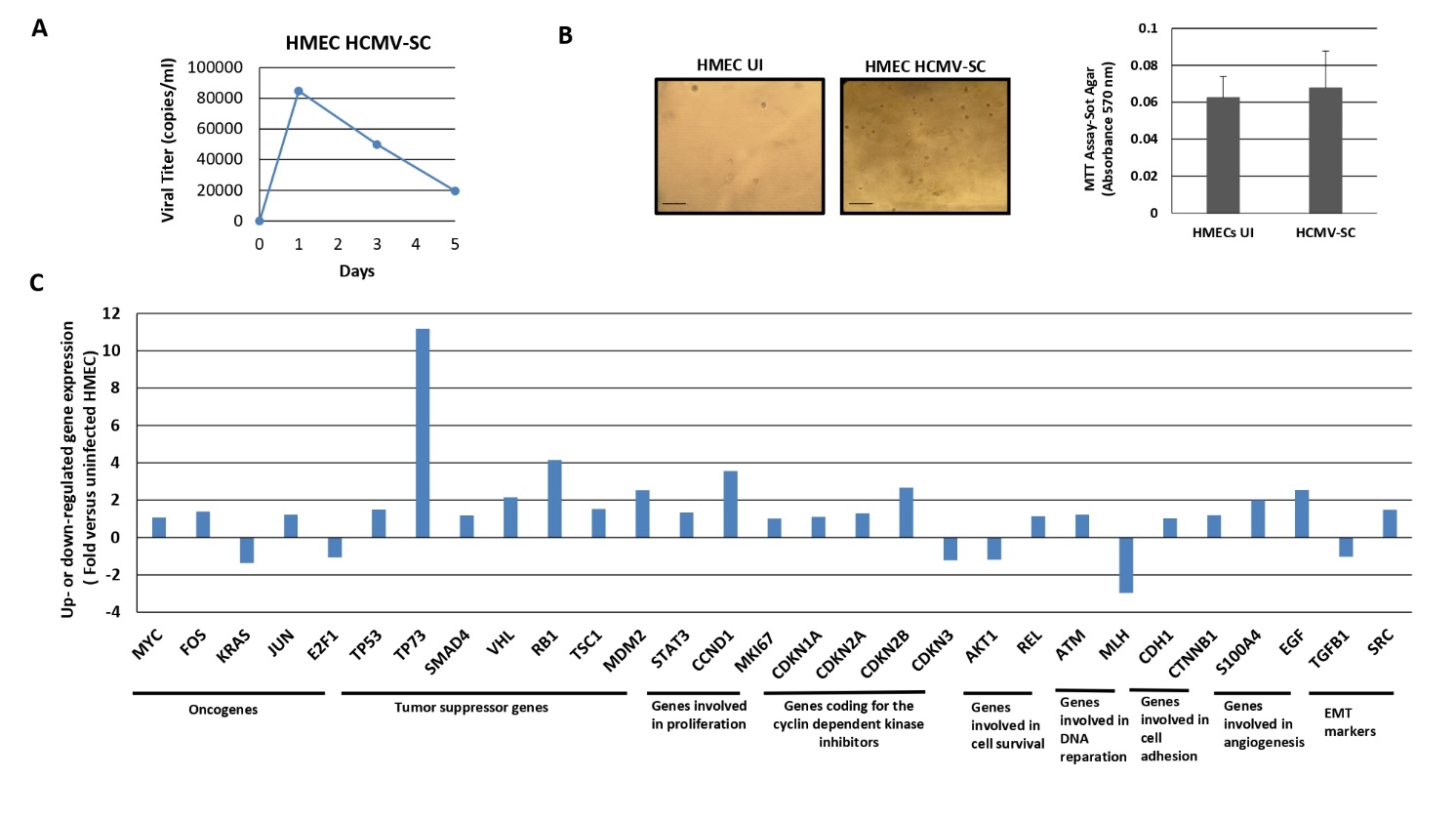


**Supplementary Figure 2.** Viral growth, soft agar colony formation and transcriptome analysis of HMECs infected with HCMV-SC. **(A)** Growth kinetics of HCMV-SC in HMECs up to day 5 post-infection as measured by IE1 qPCR in culture supernatants. **(B)** Soft-agar colony formation in HMECs seeded with HCMV-SC. **(C)** Transcriptome analysis of several genes in HMECs infected with HCMV-SC. Results are representative of two independent experiments.


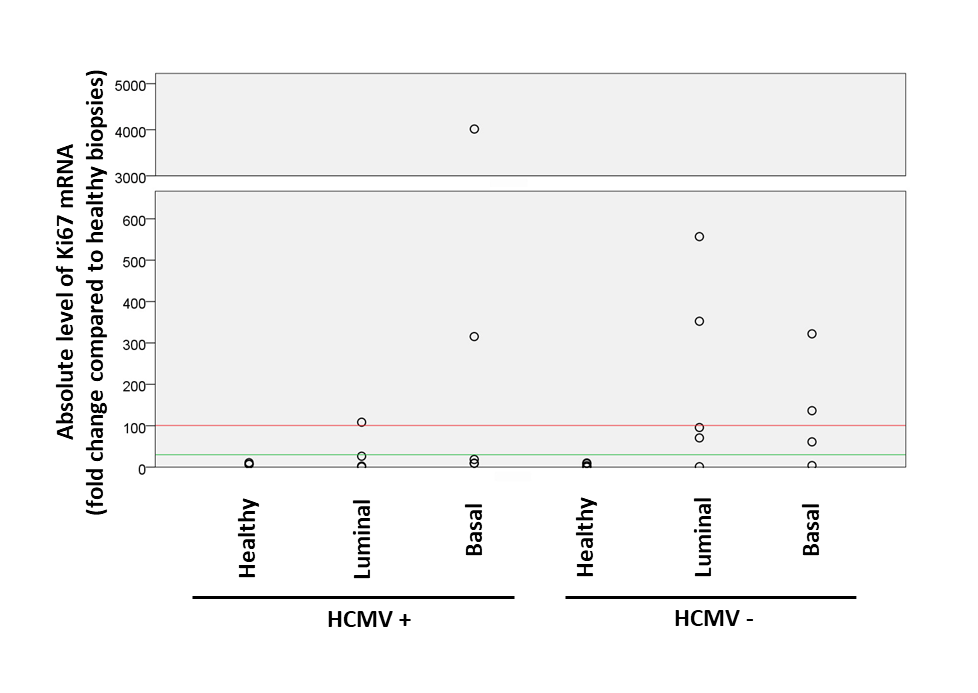


**Supplementary Figure 3.** Scatter plot of Ki-67 mRNA expression in healthy, luminal and basal biopsies which were classified into HCMV-positive and HCMV-negative biopsies. The green horizontal line represents low Ki-67 expression cutoff (<30) while the red horizontal line represents high Ki-67 expression cutoff (>100).


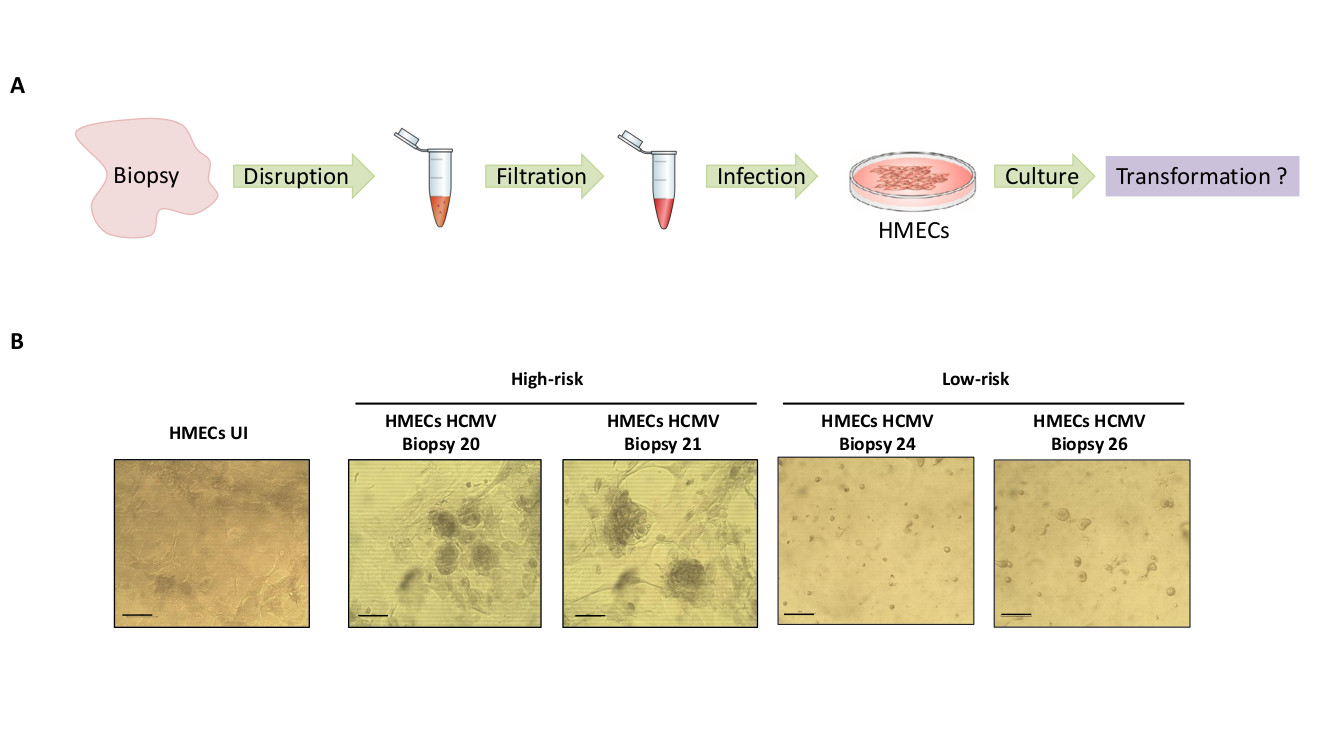


**Supplementary Figure 4.** HCMV isolation from breast cancer biopsies and subsequent infection of HMECs. **(A)** Protocol used for the isolation of HCMV strains from biopsies and infection of HMECs cells. **(B)** Pictures of uninfected HMECs and HMECs infected with high-risk and low-risk HCMV strains isolated from biopsies after 60 days of culture. Observed under inverted light-microscope, scale bar represents 100µm.

**
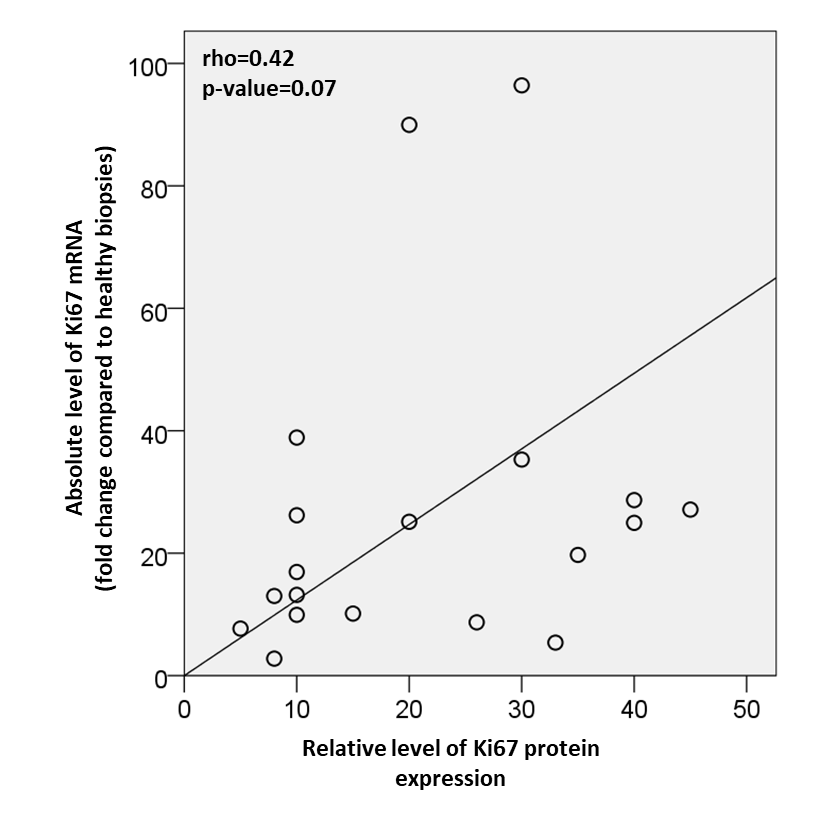
**

**Supplementary figure 5.** Scatter plot showing the correlation between Ki-67 mRNA expression and Ki-67 protein expression in breast cancer biopsies. Correlation was calculated using Spearman’s correlation test.
